# Voie antérieure transversale dans l'ostéosynthèse d'une fracture type III du processus coronoïde chez un adolescent: à propos d'un cas et revue de literature

**DOI:** 10.11604/pamj.2015.20.165.6077

**Published:** 2015-02-23

**Authors:** Amine Belmoubarik, Karim Ahed, Marouane Abouchane, Mohamed Amine Mahraoui, Yassir Elandaloussi, Ahmed Reda Haddoun, Mohamed Nechad

**Affiliations:** 1 Centre Hospitalier Universitaire Ibn Rochd, Casablanca, Maroc

**Keywords:** Processus coronoïde, fracture, abord antérieur transversal, coronoid process, fracture, transversal anterior route

## Abstract

Les auteurs rapportent une observation rare d'un jeune adolescent de 17 ans qui a présenté une fracture du processus coronoïde du coude survenue suite à un accident de sport. Il s'agit d'une observation décrivant un abord particulier par voie antérieure transversale permettant un contrôle direct et vissage en compression du fragment déplacé; l’évolution radio clinique était satisfaisante. Nous discuterons à la lumière de la littérature, notre attitude thérapeutique, et l’évolution de ce cadre nosologique à travers l'analyse de cette observation.

## Introduction

La fracture du processus coronoïde n'est pas fréquente et représente moins de 4% de l'ensemble des fractures du coude. Elle est rarement isolée, car souvent associée aux luxations postérieures du coude. De ce fait, il s'agit d'une lésion qui peut compromettre la stabilité du coude. Son traitement relève dans certains cas d'une ostéosynthèse et plusieurs voies d'abords peuvent satisfaire cette éventualité avec pour chacune des avantages et des inconvénients. Notre observation décrit le cas d'une fracture déplacée du processus coronoïde pour laquelle a été réalisé un abord antérieur transversal transbrachial permettant un vissage direct en compression avec des résultats radio cliniques et fonctionnels satisfaisants.

## Patient et observation

**Figure 1:**
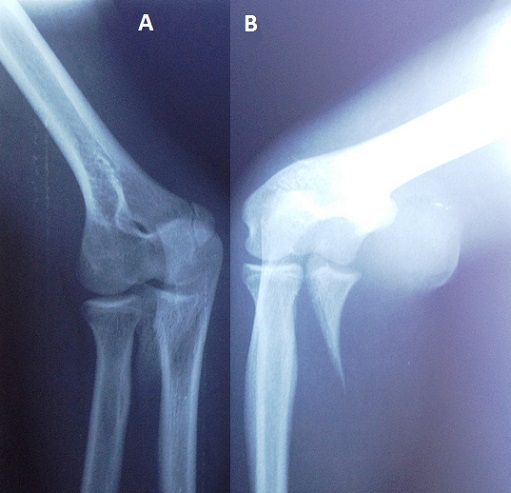
(a, b) radiographie du coude gauche de face, profil et en incidence oblique: fracture du processus coronoïde classée type III selon Regan et Morrey

**Figure 2:**
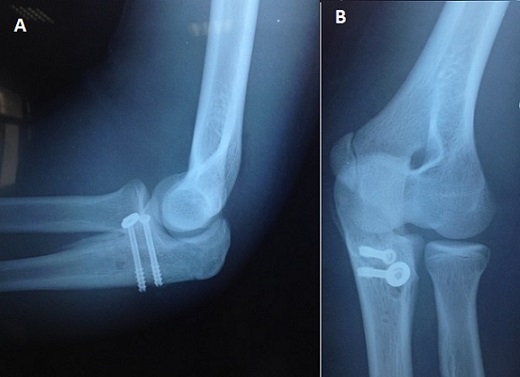
(a, b) contrôle radiographique post-opératoire immédiat: ostéosynthèse par 2 vis canulées

**Figure 3:**
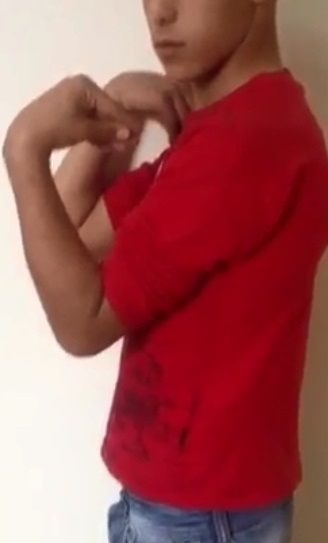
Aspect clinique post opératoire

C'est un jeune adolescent de 17 ans, de sexe masculin, droitier, étudiant, ramené aux urgences suite à une chute au sport avec réception sur la paume de la main gauche, coude en extension, avec notion de craquement ressenti par le patient au moment de la chute à l'origine d'une luxation du coude spontanément réduite. L'examen clinique trouve une impotence fonctionnelle totale, douleur, œdème important du coude, une dermabrasion étendue de la face interne du coude gauche sans déficits vasculo-nerveux associés. La radiographie du coude en incidence de face et de profil montre une fracture déplacée du processus coronoïde gauche classée type III selon Regan et Morrey, (Figure 1), Le patient a été opéré, 4 jours après son admission, sous anesthésie générale, par voie d'abord antérieure transversale du coude, le tendon du long biceps a été récliné en dehors, les fibres du brachial antérieur discisées longitudinalement, le nerf médian et pédicule huméral en dedans, nous réduisons dès lors le fragment déplacé et réalisons une ostéosynthèse en compression par deux vis canulées (Figure 2). Un drainage de la plaie retirée après 48h. Devant la stabilité du montage et l'absence de lésions associées, le membre supérieur gauche a été immobilisé par une attelle postérieure brachio-antébrachiale pendant quinze jours. L'auto rééducation commencée après ablation du plâtre et fils, a permis la récupération d'un coude stable, mobile, indolore avec légère diminution de l'amplitude de flexion à 120° par rapport au coude droit (Figure 3). Le patient était satisfait au recul de deux mois. On n'a déploré aucune complication infectieuse, vasculaire ou nerveuse au niveau du coude dont la rééducation a été conduite jusqu'au dernier recul.

## Discussion

La fracture du processus coronoïde, lésion rare [1], peut être isolé mais le plus souvent associée à une luxation postérieure du coude ou à un fracas épiphyso-métaphysaire de l'ulna. Classiquement le traitement souvent reste fonctionnel. Le diagnostic est aisément posé sur des radiographies standards du coude de face et de profil, associées à un cliché oblique externe afin de mieux apprécier la lésion. Le traitement orthopédique nous permet d'obtenir de bons à très bons résultats y compris pour des fractures associées à une instabilité, ou consécutives à des luxations. Les résultats du traitement orthopédique sont à ce jour bons ou très bons pour des fractures emportant moins de 25% de la hauteur du processus. Le résultat fonctionnel semble lié à la taille du fragment fracturaire. Selon Guiberteau et Al [2], les patients présentant un fragment inférieur à 25% ont des résultats excellents, peu importe l'existence initiale d'une laxité en varus forcé. Dès lors on peut s'interroger sur l'utilité des radiographies de stress et du scanner avec reconstructions 3D dans cette situation. En revanche pour les fragments supérieurs à 25%, les résultats ne sont que bons et moyens. Ce sont chez ces patients que nous retrouvons instabilité et arthrose. Cela laisse donc penser que le traitement chirurgical aurait été préférable. En effet, si l'on se réfère à Doornberg [3], les patients ayant bénéficié d'un traitement chirurgical présentent pour la plupart des résultats excellents. Ces résultats nous permettent de rappeler l'importance de la classification de Regan et Morrey [4] qui tient compte de la taille du fragment, critère absent de la classification d'Odriscoll [5]. C'est pourquoi, à notre avis, ces deux classifications doivent être associées pour mieux évaluer la conduite à tenir, les résultats fonctionnels semblant fortement liés à la taille de ce fragment. Dans notre observation, notre patient présente un résultat très bon, bien que le traitement par vissage dont il a bénéficié soit décrit par Ring [6] comme un traitement inadapté, la référence étant la fixation par plaque à contrefort. Le traitement chirurgical préconise le recours aux voies antérieures plutôt qu'aux postérieures qui n'offrent pas de contrôle sur le fragment fracturaire et ne permettent qu'un vissage en rappel à stabilité moindre contrairement à l'abord antérieur. Concernant ces voies d'abord antérieures, la revue de littérature n'a retrouvé que le mini abord antérieur longeant le bord médial du tendon du biceps sur un longueur de 3cm, mais devant le déplacement important du fragment fracturaire et sa grande taille, le choix d'un abord permettant d'avoir un large jour sur le processus coronoïde et de faciliter la réduction et l'ostéosynthèse en compression de ce même fragment, a été donc décidé de réaliser un abord antérieur transversal médian au niveau du pli de flexion du coude, voie qui a permis de répondre à ces exigences, permettant ainsi de mener une réduction sous contrôle de la vue et synthèse du gros fragment en position anatomique par vissage direct en moyennant 2 vis canulées d'Asnis 3,5 mm avec rondelle ce qui a conféré à ce montage une stabilité optimale.

**Figure 4:**
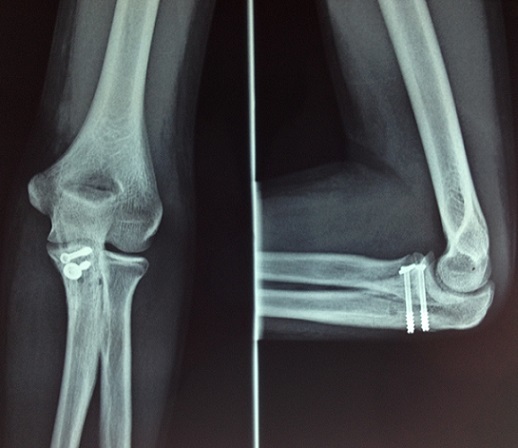
(a, b) radiographie du coude gauche de face et de profil: consolidation du processus coronoïde au recul de deux mois

**Figure 5:**
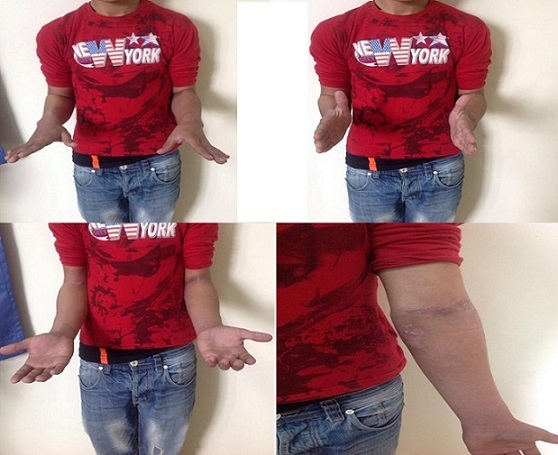
Mobilités post opératoires du coude

Au recul de deux mois: la consolidation obtenue (Figure 4), le coude est indolore, la flexion est légèrement diminuée par rapport au coude droit, les mobilités du coude sont: extension à 0°; flexion à 120°, pronosupination à 0-180° (Figure 5). Le malade a été satisfait du résultat. Ces résultats évalués selon le score fonctionnel de Mayo Elbow Performance Index (MEPI), étudiant douleur, mobilité, stabilité et fonction trouve un résultat excellent avec un score > 90 points. A la fin, nous pensons que, bien qu'il s'agisse d'une fracture résultant d'un mécanisme particulier, sa prise en charge est fortement liée à la taille du fragment et du déplacement. Cela reste cependant à confirmer par de plus grandes séries et avec plus de recul afin de ne pas sous-évaluer les risques d'arthrose huméro-ulnaire et de mieux cerner les indications chirurgicales.

## Conclusion

Le traitement chirurgical des fractures de l'apophyse coronoïde par un abord antérieur par dissociation longitudinale du muscle brachial nous parait un progrés. Par cette voie, simple et non délabrante, le vissage direct, plus stable, apparaît aisé tout en permettant une récupération fonctionnelle rapide et satisfaisante.
